# Micro-biological degradation and transformation of dissolved organic matter following continuous cropping of tobacco

**DOI:** 10.3389/fmicb.2024.1319895

**Published:** 2024-01-26

**Authors:** Peng Chen, Lei Wang, Wei-hua Li, Xiu-xia Zhang, Huan-huan Gao, Xian-hong Zhou, Qian-ying Zhuang, Jian Li, Xing-yue Li, An-sheng Zhang

**Affiliations:** ^1^Key Laboratory of Natural Enemies Insects, Ministry of Agriculture and Rural Affairs, Shandong Provincial Engineering Technology Research Center on Biocontrol of Crop Diseases and Insect Pest, Institute of Plant Protection, Shandong Academy of Agricultural Sciences, Jinan, China; ^2^College of Plant Protection, Shandong Agricultural University, Tai’an, China; ^3^School of Environmental Science and Engineering, Tianjin University, Tianjin, China; ^4^Institute of Plant Protection, Sichuan Academy of Agricultural Sciences, Chengdu, China

**Keywords:** DOM, molecular composition, continuous cropping, bacterial community, tobacco

## Abstract

In recent years, the problems associated with continuous cropping (CC) that cause soil degradation have become increasingly serious. As a key soil quality property, dissolved organic matter (DOM) affects the circulation of carbon and nutrients and the composition of bacterial communities in soil. However, research on the changes in the molecular composition of DOM after CC is limited. In this study, the soil chemical properties, DOM chemical diversity, bacterial community structure, and their interactions are explored in the soil samples from different CC years (CC1Y, CC3Y, CC5Y, and CC7Y) of tobacco. With increasing CC year of tobacco, most of the soil chemical properties, such as total carbon, total nitrogen and organic matter, decreased significantly, while dissolved organic carbon first decreased and then increased. Likewise, the trends of DOM composition differed with changing duration of CC, such as the tannin compounds decreased from 18.13 to 13.95%, aliphatic/proteins increased from 2.73 to 8.85%. After 7 years of CC, the soil preferentially produced compounds with either high H/C ratios (H/C > 1.5), including carbohydrates, lipids, and aliphatic/proteins, or low O/C ratios (O/C < 0.1), such as unsaturated hydrocarbons. Furthermore, core microorganisms, including *Nocardioides*, *wb1-P19*, *Aquabacterium*, *Methylobacter*, and *Thiobacillus*, were identified. Network analysis further indicated that in response to CC, *Methylobacter* and *Thiobacillus* were correlated with the microbial degradation and transformation of DOM. These findings will improve our understanding of the interactions between microbial community and DOM in continuous cropping soil.

## 1 Introduction

Cultivated land is an important component of agricultural ecosystems and provides essential land-related resources (He et al., 2023). In recent years, the quality and ecology of cultivated land have attracted attention from the government. Agricultural land degradation is often affected by the loss of soil fertility and biodiversity, which is commonly caused by unsustainable agricultural land management practices, such as long-term monoculture cropping (Chen S. et al., 2018; Chen et al., 2020; Yu and Jesús, 2021). In China, because of the limited area of cultivated land, the same crop is often continually grown in the same field without interruption, which typically leads to the continuous cropping (CC) obstacle. Generally, the CC obstacle manifests as a decline in soil quality and crop growth, and seriously restricts the development of crop farming (Ku et al., 2022). Tobacco (*Nicotiana tabacum* L.) has been used as a model organism as it is an important crop for millions of Chinese farmers. CC leads to poor seedling growth and yield reduction, about 60% of tobacco in China is grown in CC soil, causing economic losses of up to 4 billion (Niu et al., 2017; Chen S. et al., 2018; Dong et al., 2022).

The plant rhizosphere soil environment is changeable, and long-term monoculture significantly disturbs many soil factors, e.g., nutrients and microbial communities (Chen et al., 2020). Dissolved organic matter (DOM), which can directly reflect organic matter (OM) stored in the soil, is a key soil quality property (Al-Graiti et al., 2022). DOM is defined as a ubiquitous mixture of organic compounds in terrestrial ecosystems, the mobility of which affects the circulation and distribution of both carbon and nutrients in soil (Fox et al., 2018; Hu et al., 2021). Based on the H/C and O/C ratio of DOM molecules, they can be divided into seven types of compounds, including lipids (O/C = 0–0.3 and H/C = 1.5–2.0), aliphatic/proteins (O/C = 0.3–0.67 and H/C = 1.5–2.2), lignin/carboxylic rich alicyclic molecules (CRAM-like) (O/C = 0.1–0.67 and H/C = 0.7–1.5), carbohydrates (O/C = 0.67–1.2 and H/C = 1.5–2.3), unsaturated hydrocarbons (O/C = 0–0.1 and H/C = 0.5–1.5), condensed aromatics (O/C = 0–0.67 and H/C = 0.1–0.7) and tannins (O/C = 0.67–1.2 and H/C = 0.5–1.5) (Ohno et al., 2010; Che et al., 2020a). Additionally, various researchers have shown that the aliphatic/proteins DOM has poor stability and are decomposed faster, while lignin/CRAM-like DOM is degraded into aromatic compounds with lower H/C (Kaiser and Kalbitz, 2012). In a naturally stable ecosystem, such as a native forest, DOM is generally composed of a specific soil organic matter (SOM) content and composition (Al-Graiti et al., 2022). However, due to the use of frequent tillage, a considerable part of SOM is lost, resulting in the biodegradation and rapid mineralization of SOM (Virginia et al., 2022). Due to the biodegradation of SOM occurring in water-soluble phases, DOM can directly reflect most of the characteristics of SOM (Che et al., 2020a). Our previous study has shown that the SOM content in soil changes significantly under CC (Li et al., 2018; Chen et al., 2020). The depletion of organic carbon under CC has led to a decline in crop yields (Sun et al., 2011). However, the changes in the molecular composition of DOM after CC are not well understood at present.

It is well-known that soil microorganisms play significant roles in many key processes of the soil ecosystem, such as nutrition cycling and SOM turnover (Zhang J. et al., 2020). Previous studies have demonstrated the effects of the microbial community on the decomposition and transformation of organic compounds (Zhong et al., 2017; Che et al., 2020a). The growth and activity of soil microbes are closely associated with the DOM composition of the soil (Bai et al., 2019; Zheng et al., 2019). For example, DOM provides various soluble organic substrates to soil microbial communities, such as aliphatic compounds (Bourbonniere and Creed, 2005). Moreover, the generation of DOM molecules in soil is accompanied by microbial processing (Li et al., 2019). Previous research showed that *Firmicutes* can accelerate the formation of small-molecule DOM (Che et al., 2020a). In contrast, *Prosthecobacter*, *Paenalcaligenes*, and *Solibacillus* are mainly involved in the transformation and stabilization of DOM (Zhu et al., 2019). Our previous research has shown that long-term monoculture significantly affects the structure and diversity of the soil microbial community (Li et al., 2018; Chen et al., 2020). As previously mentioned, the interactions between the chemodiversity of DOM and microbial communities revealed by network analyses should be updated with more in-depth analyses or validated.

In the present study, samples of soil that were subjected to different durations of CC (1, 3, 5, and 7 years) were used to evaluate the micro-biological degradation and transformation of DOM. The objectives of the study were: (1) to explore the changes in soil basic chemical properties, DOM, and soil microbial communities at different CC soil; and (2) to evaluate micro-biological degradation and transformation pathways of DOM after different durations of CC. The knowledge obtained from this study provides useful information for the occurrence factors of the CC obstacles of tobacco.

## 2 Materials and methods

### 2.1 Site description and sampling

This experiment was performed in Jiayue Town (36°02′20″N, 119°12′73″E), Zhucheng City, Shandong Province, China. The mean annual temperature in this area is approximately 13.2°C, the mean annual precipitation is 741.8 mm, and the mean annual evapotranspiration is 1,677.5 mm. According to the World Reference Base, the soil is classified as Typic Hapli-Ustic Argosols (Chesworth et al., 2008).

The tobacco variety used in this experiment was *Nicotiana tabacum* L. cultivar ‘Zhongyan100.’ The soil samples included four groups: CC with tobacco for 1 year (CC1Y), 3 years (CC3Y), 5 years (CC5Y), and 7 years (CC7Y). All fields were subjected to the same agricultural management practices, which included the following basic fertilizer applications: 450 kg/ha NPK compound fertilizer (N:P:K = 10:10:20), 450 kg/ha potassium sulfate (K_2_O ≥ 52%), and 75 kg/ha diammonium phosphate (N + P_2_O_5_ ≥ 64%).

In this study, four treatment groups were used (i.e., CC1Y, CC3Y, CC5Y, and CC7Y), totaling 60 rhizosphere soil samples (5 samples × 3 biological replications × 4 treatments). The soil samples were taken from soils that had experienced 1, 3, 5, or 7 years of CC of tobacco in June 2021. The tobacco roots were carefully removed from the soil at a depth of 0–30 cm. For this study, rhizosphere soil was mainly collected, located within 2 mm of the root surfaces. Each sample consisted of three replicates. Each replicate contains 5 samples, which were collected from different tobacco fields by 5-point sampling. Then, these samples were mixed in an aseptic bag to form a replicate. The samples were immediately transported to the laboratory and divided into two parts: one was dried at 60°C for analyses of soil chemical properties, and the other was stored at −80°C in the freezer.

### 2.2 Soil chemical properties

The soil pH-H_2_O was measured by a pH meter with a glass electrode (FE20-Five Easy Plus^™^, Switzerland). Total carbon (TC) and total nitrogen (TN) in soil samples were measured by the Costech ECS 4010 element analyzer (Costech Analytical Technologies, Inc., Valencia, CA, USA). Soil OM was assayed using the vitriol acid potassium dichromate oxidation method (Sparks et al., 1996).

### 2.3 DOM extraction and ESI-FT-ICR-MS analysis

DOM extraction from the obtained soil samples was conducted as described by Yuan et al. (2012). In brief, the fresh soil sample was mixed with distilled water (1,10, w/v ratio) by shaking for 24 h at 20°C in an incubator. The suspensions were centrifuged at 15000 r/min for 15 min, and then filtered through a 0.45-μm filter membrane. The extracted DOM aqueous solution was stored at 4°C until further analysis.

The content of dissolved organic carbon (DOC) was determined by a TOC-VCPH analyzer (Shimadzu, Japan). DOM in soil samples was analyzed by ESI-FT-ICR-MS, following the method of Li et al. (2022). Briefly, the pH of the extracted DOM was adjusted to 2 using HCl (pH = 1, MS grade), and all collected samples were extracted by Varian Bond Elute PPL cartridges (200 mg, 3 mL^−1^) to remove salts. All purified DOM samples were measured with the 15.0 T Bruker Solari X FT-ICR-MS (Bruker, Billerica, MA, USA) equipped with an ESI ion source. DOM samples were detected at negative ionization mode. The examination area ranged from m/z 200–1,000, and the ion accumulation time was adjusted to 0.2 s. 4 M words data were recorded per broadband mass scan, where each mass spectrum was scanned 100 times to enhance the signal-to-noise ratio and dynamic range. Molecular formula assignment calculator software was used to calculate all mathematically possible formulas for all ions with a signal-to-noise ratio ≥ 4 according to stringent criteria. Molecular formulas with elemental combinations of ^12^C_(0-x)_, ^1^H_(0 − y)_, ^16^O_(0 − z)_, ^14^N_(0–3)_ and ^32^S_(0–1)_ were assigned to peaks. The elemental ratios of H/C < 2.4 and O/C < 1.2 were detected and the data were further analyzed according to the methods of Lv et al. (2018).

### 2.4 Soil bacterial diversity

Total soil DNA was extracted from fresh soil (500 mg) using a PowerSoil^®^ DNA Isolation Kit (MO Bio Laboratories, San Diego, CA, USA) according to the manufacturer’s protocol. The V3–V4 region of the 16S bacterial rRNA gene was amplified using the universal primer pair 338F (5′-GTACTCCTACGGGAGGCAGCA-3′) and 806R (5′-GTGGACTACHVGGGTWTCTAAT-3′) to identify soil bacterial communities (Kim et al., 2010). The 16S rRNA gene was amplified by polymerase chain reaction before sequencing. The polymerase chain reaction was performed in a total volume of 50 μL, including 2 μL of template DNA, 5 μL of 10 × buffer, 2 μL of forward primer (10 μM), 2 μL of reverse primer (10 μM), 4 μL of dNTPs (2.5 μM), 0.3 μL of DNA polymerase (2.5 U μL^−1^), and 34.7 μL of ddH_2_O. Reaction conditions were 5 min at 95°C, followed by 30 cycles of 30 s at 95°C, 30 s at 56°C, 40 s for elongation at 72°C, and a final extension at 72°C for 10 min followed by a hold at 10°C.

The purified DNA was quantified via a QuantiFluor^™^-ST (Promega, USA) and subsequently sequenced (PE300) on an Illumina MiSeq platform (Illumina Inc., USA) by Shanghai Majorbio Bio-pharm Technology Co., Ltd. Noisy amplicon sequences (i.e., low-quality sequences) were removed using the Precluster tool of the Quantitative Insights into Microbial Ecology package (ver. 1.2.1). Operational taxonomic units were defined based on 97% similarity level and clustered by UPARSE (ver. 7.1) (Edgar, 2013). Chimeric sequences were identified and removed by UCHIME (Edgar et al., 2011). Representative sequences of each operational taxonomic unit were taxonomically classified by the Silva and Unite database (Quast et al., 2012).

### 2.5 Data analysis

Biodiversity data were analyzed on the free online platform of Majorbio I-Sanger Cloud Platform.^1^ The relationship among DOM molecular composition, chemical properties, and bacterial genera was analyzed using Networkx (ver. 1.11). Graphics were drawn by Origin Pro 2022 software (OriginLab, USA). The data were analyzed by one-way ANOVA (Duncan test; *p* < 0.05) using SPSS 20.0 (SPSS, Chicago, IL, USA). A *p*-value < 0.05 was regarded to indicate significant differences.

## 3 Results and discussion

### 3.1 Analysis of soil chemical properties following CC

**Figure 1:**
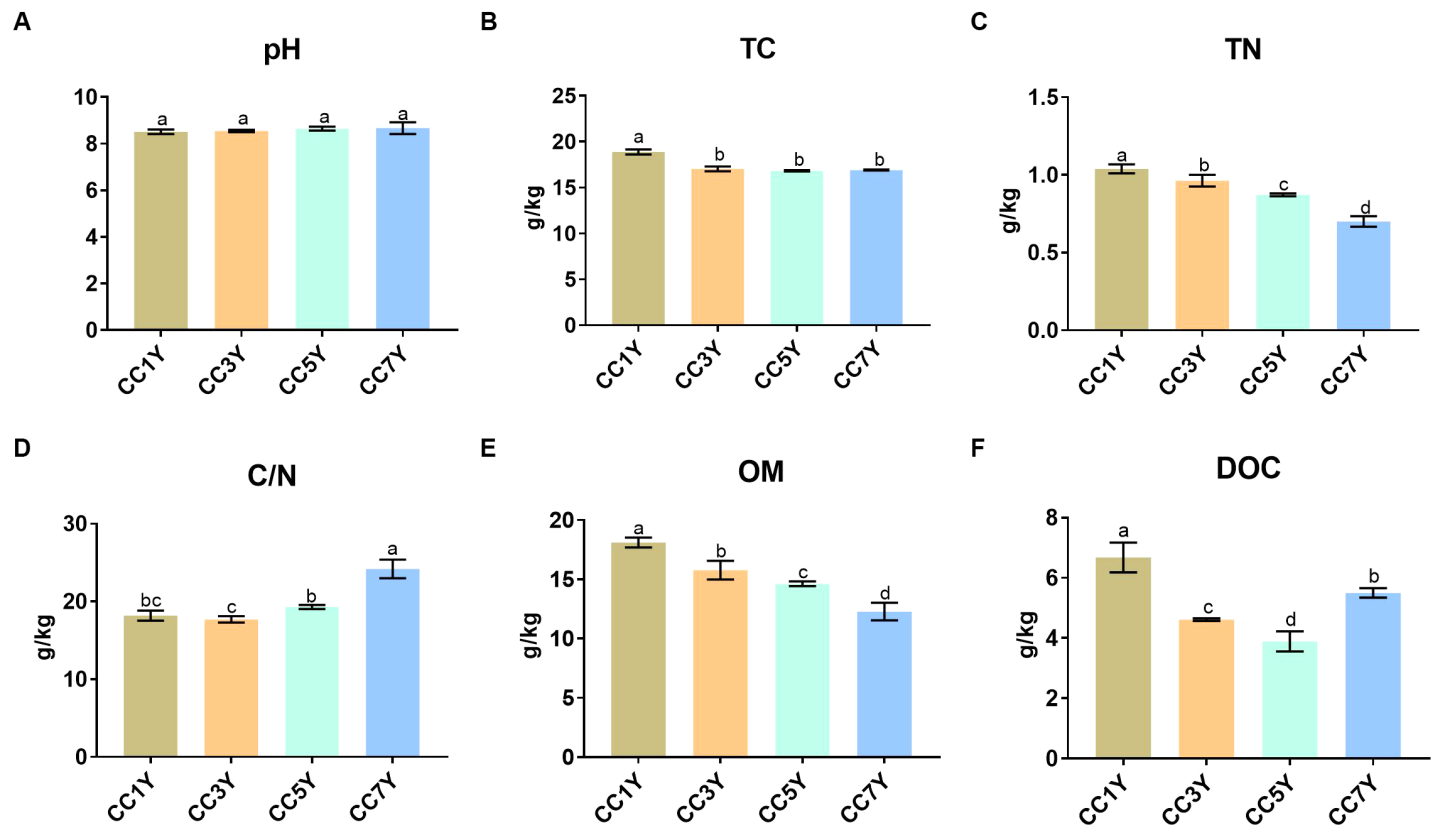
Changes in soil chemical properties after different durations of CC: **(A)** pH, **(B)** total carbon (TC), **(C)** total nitrogen (TN), **(D)** C/N (TC/TN), **(E)** organic matter (OM), and **(F)** dissolved organic carbon (DOC). The letters indicate significant differences among different years of CC (calculated according to analysis of variance: Duncan test; *p* < 0.05).

The effects of different CC durations on soil chemical characteristics are shown in Figure 1. The soil pH did not differ significantly in different CC years (Figure 1A). Compared to CC1Y, the TC content was significantly lower in CC3Y, CC5Y, and CC7Y (Figure 1B). The concentrations of TN and OM gradually decreased with increasing duration of CC (Figures 1C,E). Soil chemical properties were negatively affected by the continuous-cropping obstacle, which is consistent with the findings of Chen S. et al. (2018). This phenomenon was attributed to the unbalanced nutrient supply and the preference for nutrient absorption during crop cultivation (Li et al., 2023). Soil OM plays a vital role in the provision of nutrients for plants and microbes; thus, the decreasing OM content indicated that soil fertility decreased with increasing duration of CC (Obalum et al., 2017; Chen S. et al., 2018). In contrast, the C/N increased gradually in CC5Y and CC7Y (Figure 1D). This is due to the rapid decrease of soil TN content, as the soil C/N ratio showed an upward trend after 5 years of CC. The DOC contents followed a significant decreasing trend during the early stage of CC and increased slightly after 7 years of CC (Figure 1F), which is consistent with Li et al. (2021), from at least 5-year continuous cropping stands onwards. Furthermore, previous study found an opposite phenomenon during the process of vegetation restoration, which indicated that the restoration of continuous cropping soil might be necessary to increase the soil carbon content (Hu et al., 2021).

### 3.2 Molecular characterization of DOM in CC soil

**Figure 2:**
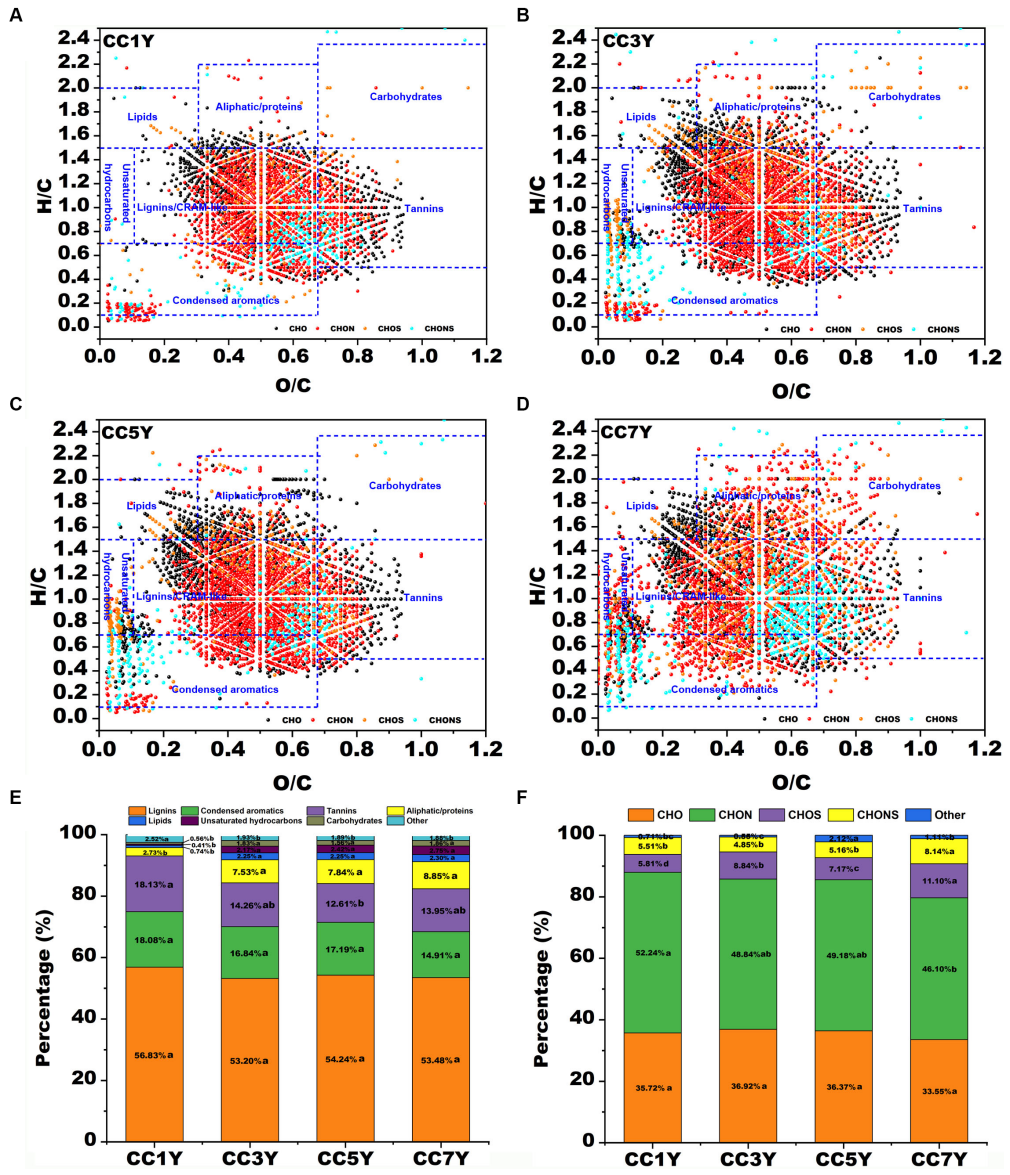
Compositional changes of dissolved organic matter (DOM) after different durations of CC. **(A–D)** Van Krevelen diagram of the four major subcategories according to different durations of CC: **(A)** DOM in CC1Y, **(B)** DOM in CC3Y, **(C)** DOM in CC5Y, and **(D)** DOM in CC7Y. The areas within crossed lines indicate different biochemical classes of identified DOM formulas. **(E)** The bar graph displays the percentages of the four major subcategories for different durations of CC. **(F)** The bar graph displays the identified major compounds for different durations of CC.

The molecular characteristics of DOM disclose the unique molecular composition of soils after different durations of continuous tobacco production. A total of 3,922, 4,930, 4,926, and 5,740 molecules were identified from CC1Y, CC3Y, CC5Y, and CC7Y samples, respectively. Van Krevelen diagrams were used to display the molecular characteristics of DOM in different soil samples (Figure 2). According to H/C and O/C ratios, the DOM formulae in the van Krevelen diagrams could be grouped into seven primary regions (Yuan et al., 2017). The relative abundance of major compound classes in soil DOM clearly changed with extended CC time. As shown in Figures 2A–D, the points corresponding to soil DOM on CC1Y are mainly distributed in the lignins/CRAM-like and tannin regions, while the points on the CC7Y showed a dispersed distribution in the lignins/CRAM-like, condensed aromatics, aliphatic/proteins, unsaturated hydrocarbons, and lipids regions. The change trends of the seven biochemical compounds in the DOM with increasing duration of continuous tobacco cropping were shown in Figure1E. The points corresponding to the soil DOM are mainly distributed in the CRAM-like regions (53.20–56.83%) (Figure 2E). This result is consistent with the water-soluble lignin released by terrestrial plants (Zhang et al., 2023). In the soil of tobacco plantations, tannins decreased from 18.13 to 12.61% after 5 years CC. Moreover, the percentage of aliphatic/proteins increased significantly from 2.73 to 8.85% with increasing duration of CC. In the soil of naturally growing forests, Li et al. (2021) found that plant-derived DOM compounds, including lignins and tannins, remained stable or increased slightly, while microbially derived DOM molecules (e.g., aliphatic compounds and proteins) decreased with stand age. In the present study, the opposite results were obtained, we found that the plant-derived DOM compounds (such as tannins) decreased significantly, while microbially derived DOM molecules (i.e., aliphatic/proteins) increased with increasing duration of CC in tobacco. Studies have shown that long-term CC can increase the size of harmful microbial communities, which provide a source of microbially derived DOM molecules (She et al., 2017; Chen et al., 2020). In plants, they inhibit the invasion of harmful microorganisms by absorbing tannins from the soil (Tamara et al., 2003). Therefore, the changes in the content of soil substances caused by CC need to be counteracted by supplementing with exogenous substances. For instance, industrial-scale compost can provide sufficient tannins compounds and accelerate the bio-oxidation of protein-like substances (Che et al., 2020b).

In general, the chemical formula of natural OM was divided into four the major groups of CHO, CHON, CHOS, and CHONS (Song et al., 2018). In this study, more than 45% of all detected subcategories could be assigned to CHON groups, followed by CHO groups (33.55–36.92%), CHOS groups (5.81–11.10%), and CHONS groups (4.85–8.14%) (Figure 2F). With increasing duration of CC years, the percentages of CHON groups decreased from 52.24 to 46.10%, while the percentages of CHO did not show an obvious variation pattern from CC1Y, CC3Y, CC5Y, and CC7Y (Figure 2F). The CHON groups have the highest content in all DOM samples, indicating that many N-containing compounds were degraded and generated due to the degradation and formation of protein, or microbial biomass produced by strongly microbial activities (Valle et al., 2018; Yu et al., 2019). However, continuous cropping resulted in a decrease in percentages of CHON, which was opposite to the variation trend of water-soluble nitrogen during composting (Che et al., 2020a). On the contrary, in soil subjected to CC, the proportion of CHOS and CHONS groups increased from 5.81 to 11.10% and 5.51 to 8.14% with extended CC, respectively (Figure 2F). It has been reported that the changes of sulfur respiration microbes in soil are closely related to the content of CNOS and CHNOS groups (Li et al., 2022).

### 3.3 Molecular insight into degradation and transformation of DOM after CC

**Figure 3:**
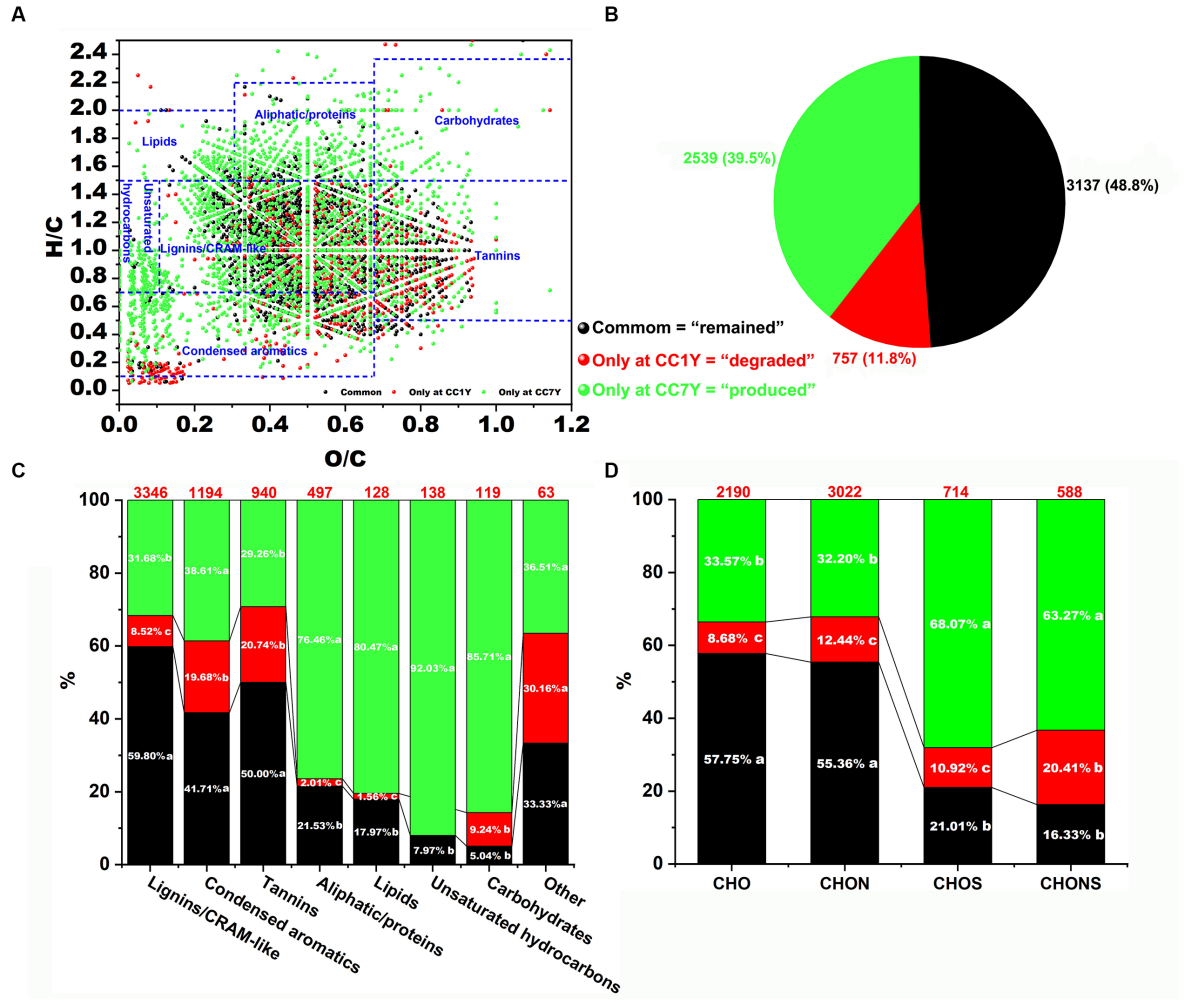
Appraisal and comparison of molecular DOM formulas at CC1Y and CC7Y. **(A)** Van Krevelen diagram comparing the remaining, degraded, and produced compounds in DOM. The areas within crossed lines indicate different biochemical classes of identified DOM formulas. **(B)** Percentages of DOM formulas identified only at CC1Y (red), common (black) at CC1Y and CC7Y, and only at CC7Y (green). **(C)** Bar chart displaying the percentages of seven identified DOM compounds. The number of DOM formulas in each class is shown above the bar. **(D)** Bar graph representing the percentages of the four subcategories. The number of DOM formulas in each group is displayed above the bar.

The distributions of degraded compounds (only at CC1Y), remaining compounds (i.e., identified at both CC1Y and CC7Y), and produced compounds (only at CC7Y) in DOM after CC are expressed as a van Krevelen diagram (Figure 3A). In this study, 48.8% of DOM molecules remained unchanged in CC7Y soil compared to CC1Y soil (Figures 3A,B). Common DOM compounds mainly contain stable or refractory DOM compounds, or compounds that were only structurally transformed but maintained the same elemental composition (Chen H. et al., 2018). Furthermore, the unique formulas (11.8%) in the CC1Y soil samples represent those degraded after 7 years of CC, whereas unique formulas (39.5%) identified in CC7Y soil samples are newly produced molecules (Figure 3B). The numbers of the degraded, remaining, and produced DOM formulas in the seven regions are presented in Figure 3C. Clearly, condensed aromatics and tannins decomposed easily after CC, whereas lignin/CARM-like were the most abundant and difficult to biodegrade compounds (Figure 3C). Many previous studies have also reached similar conclusions, namely that lignins in soil were the most refractory compounds (Chen H. et al., 2018; Che et al., 2020a). The quality of crop cultivation soil can be represented by the compounds produced after long-term CC. In this study, the compounds that were produced most abundantly were unsaturated hydrocarbons (92.03%), carbohydrates (85.71%), lipids (80.47%), and aliphatic/proteins (76.46%). These produced DOM formulas were mainly distributed in higher H/C ratios (H/C > 1.5) or lower O/C ratios (O/C < 0.1) than the decomposed compounds (Figure 3A). Large amounts of high H/C and low O/C by-products produced in CC soil could result in inputs of N and S (Durham et al., 2014; McDonough et al., 2022). The numbers of four compound subcategories are shown in Figure 3D based on their elemental composition. The CHO and CHON molecules were stable, with >55% of the formulas remaining after 7 years of CC. These findings are in accordance with previous studies, which reported that CHO and CHON represented the most refractory compounds after composting (Che et al., 2020a). CHOS formulas were the most labile compounds, and > 68% were newly produced after long term CC. This trend is matched by CHONS molecules, > 63% of which only appeared in CC7Y soil.

### 3.4 Characterization of the microbial community structure in continuously cropped soil

**Figure 4:**
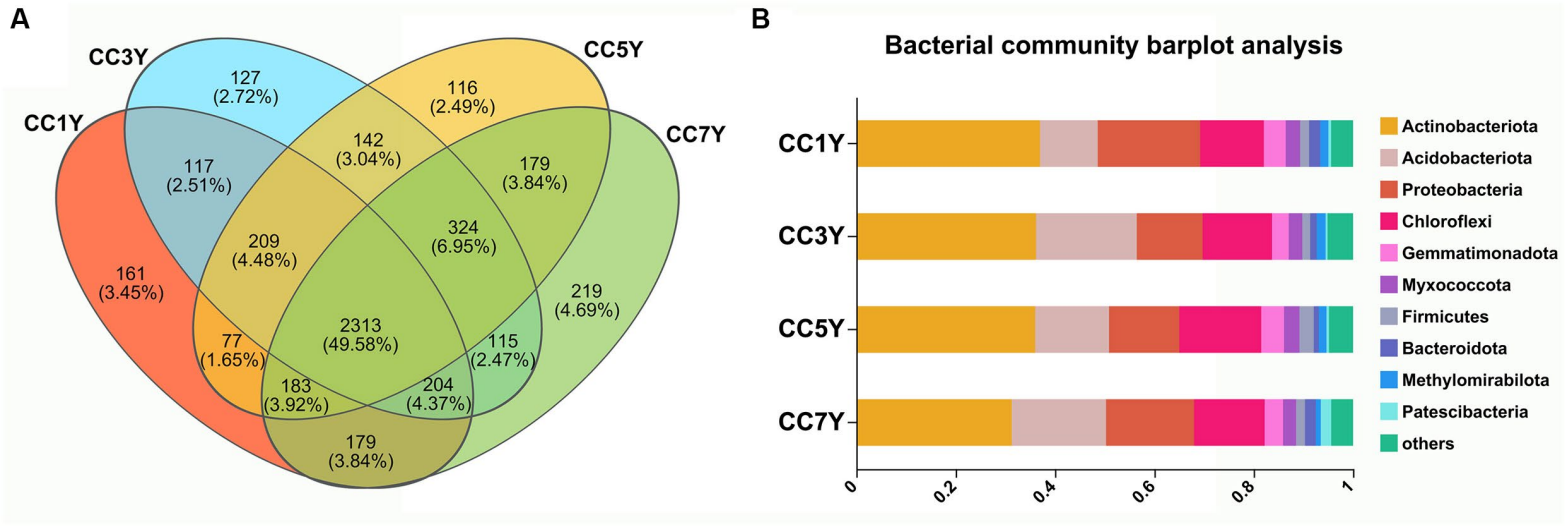
Bacterial composition and structure of soil communities after different durations of CC. **(A)** Venn diagrams of CC samples in different years. **(B)** Bacterial community histogram at the phylum level (the relative abundance >1%).

The cycling of elements in soil is closely related to the types and activities of soil microorganisms (Kronzucker et al., 1997). In this study, the bacterial alpha diversity index of soils that were subjected to different durations of CC did not change significantly (Supplementary Figure S1). However, the sobs estimator for bacterial communities significantly increased in CC7Y, indicating that the richness of soil bacteria was higher in CC7Y than in CC1Y (Supplementary Figure S1C). The bacterial taxonomic compositions of continuously cropped soil samples in different years are shown in Figure 4. The Venn Diagram shows that 49.58% (2313) of the operational taxonomic units exist in all samples (Figure 4A). At the phylum level (relative abundance >1%), the dominant bacterial community in the soil consisted of Actinobacteriota (31.30–37.00%), Acidobacteriota (11.62–20.29%), Proteobacteria (13.25–20.62%), and Chloroflexi (12.81–16.51%) (Figure 4B and Supplementary Figure S2). The relative abundances of Actinobacteriota, Acidobacteriota, and Proteobacteria did not differ significantly between different samples. Acidobacteria is an efficient colonizer of acidic terrestrial habitats. The abundance of Acidobacteria did not change significantly after 7 years CC, which was consistent with the stable soil pH (Figure 1; Belova et al., 2018). However, the increasing trend of Acidobacteria content found in this study suggests that the tobacco CC obstacle is a process of soil acidification. Furthermore, the phylum Chloroflexi had increased in CC5Y compared to CC1Y (Figure 4B and Supplementary Figure S2). According to previous reports, the increased soil nitrogen could decrease oligotrophic taxa, such as the taxonomic groups of Chloroflexi (Nie et al., 2018; Li et al., 2021). The results of this study support the above conclusion, as increased oligotrophic taxa (Chloroflexi) were found to occur with decreased soil TN in CC5Y (Figures 1, 4B and Supplementary Figure S2).

**Figure 5:**
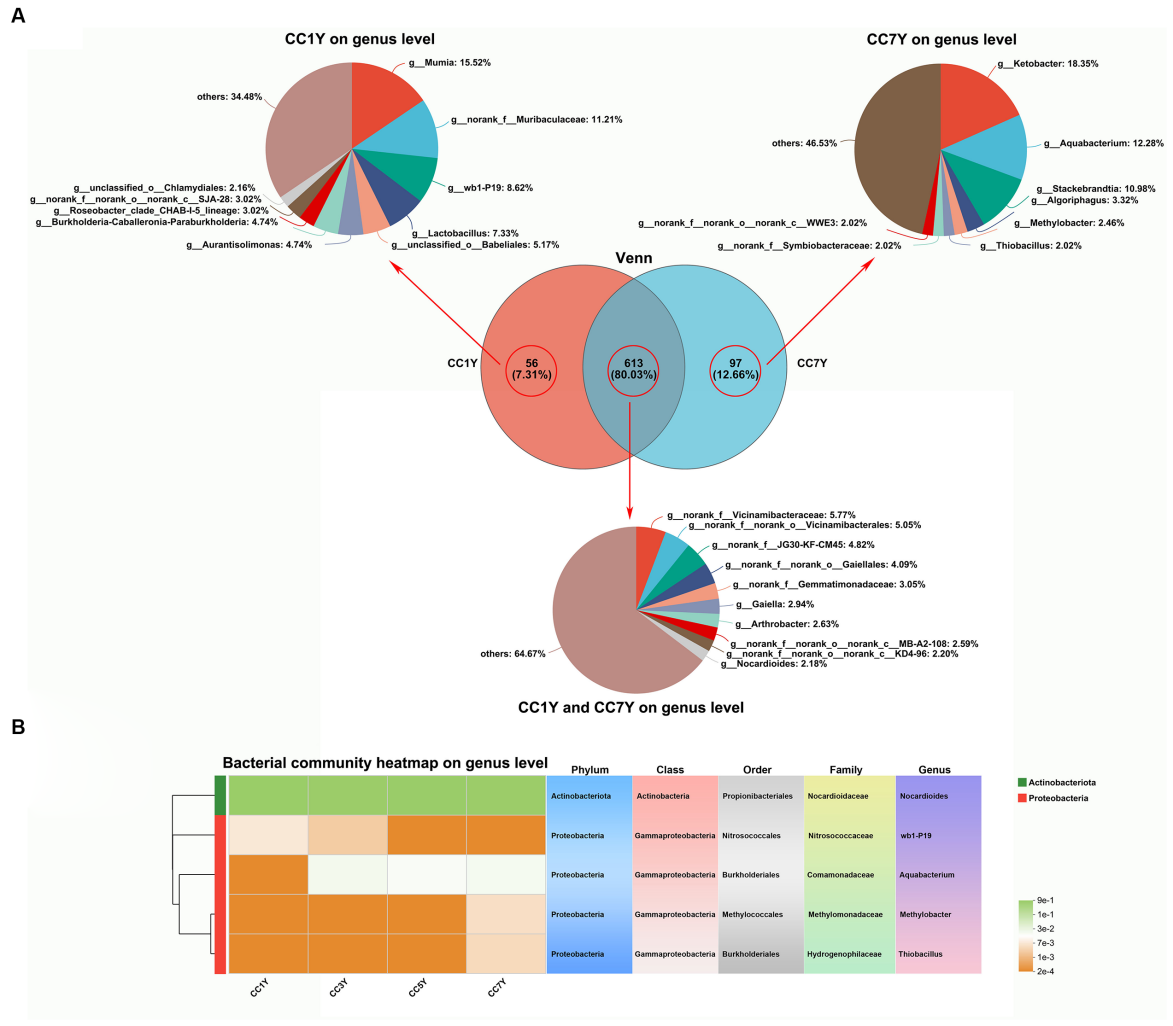
Assessment and comparison of bacterial compositions at the genus level between soil samples subjected to different durations of CC. **(A)** Venn diagrams and relative abundances of bacterial genera found only at CC1Y, commonly found at CC1Y and CC7Y, and found only at CC7Y. **(B)** Key dominant groups of bacteria in different classifications.

In the current study, the changes in genus-level bacterial taxonomic compositions were further compared between CC1Y and CC7Y. The Venn diagram shows that 613 (80.03%) genera were identified in both CC1Y and CC7Y, 7.31% (56) genera were only identified in CC1Y, and 12.66% (97) genera were only identified in CC7Y (Figure 5A). Microorganisms are the drivers of soil elemental cycling at the ecosystem scale, and the cycled elements are carbon (C), nitrogen (N), and Sulfur (S) (Spohn, 2016). In this study, the genus *Nocardioides* was found in both CC1Y and CC7Y samples, *wb1-P19* was only found in CC1Y, and *Aquabacterium*, *Methylobacter*, and *Thiobacillus* were only found in CC7Y samples (Figure 5A). *Nocardioides* (belonging to Actinobacteriota) and four other genera belonging to Proteobacteria were all dominant phyla in soil samples (Figures 4B, 5B). *Nocardioides*, *wb1-P19*, and *Aquabacterium* are mainly involved in the cycling process of nitrogen elements in soil. *Nocardioides* was reported to respond positively to nitrogen addition and could play a role in nitrogen fixation (Song et al., 2007; Huang et al., 2021). Thus, *Nocardioides* had a high relative content in all samples (Figure 5B). Nitrogen concentrations in soil systems are the origin of electron donors and acceptors for microorganisms; *wb1-P19* (belonging to Nitrosococcaceae) and *Aquabacterium* can use nitrate as an electron acceptor (Attard et al., 2010; Zhang et al., 2016; Dong et al., 2020). In the current study, *wb1-P19* was only detected in CC1Y and CC3Y, while *Aquabacterium* was detected in CC3Y, CC5Y, and CC7Y, where these participated in the cycling process of nitrogen elements (Figure 5B). Additionally, the genera *Methylobacter* and *Thiobacillus* increased significantly after 7 years of CC (Figure 5B). Methanotrophs (e.g., *Methylobacter*) play a crucial role in the oxidation process of methane (CH_4_) in soil (Zhang L. et al., 2020). Thus, 33.57% of the produced CHO compounds were closely related to *Methylobacter*. *Thiobacillus* was reported to be the driving force for the oxidation of metal sulfides in soil (Edwards et al., 2004). Thus, the readily available biosynthesis substances of S-containing compounds could be rapidly increased by *Methylobacter*. This result is in line with the finding that more than 63% of the CHOS and CHONS subcategory was produced after 7 years of CC (Figure 3D).

### 3.5 Insight into the interactions among DOM molecular composition, chemical properties, and bacterial genera

**Figure 6:**
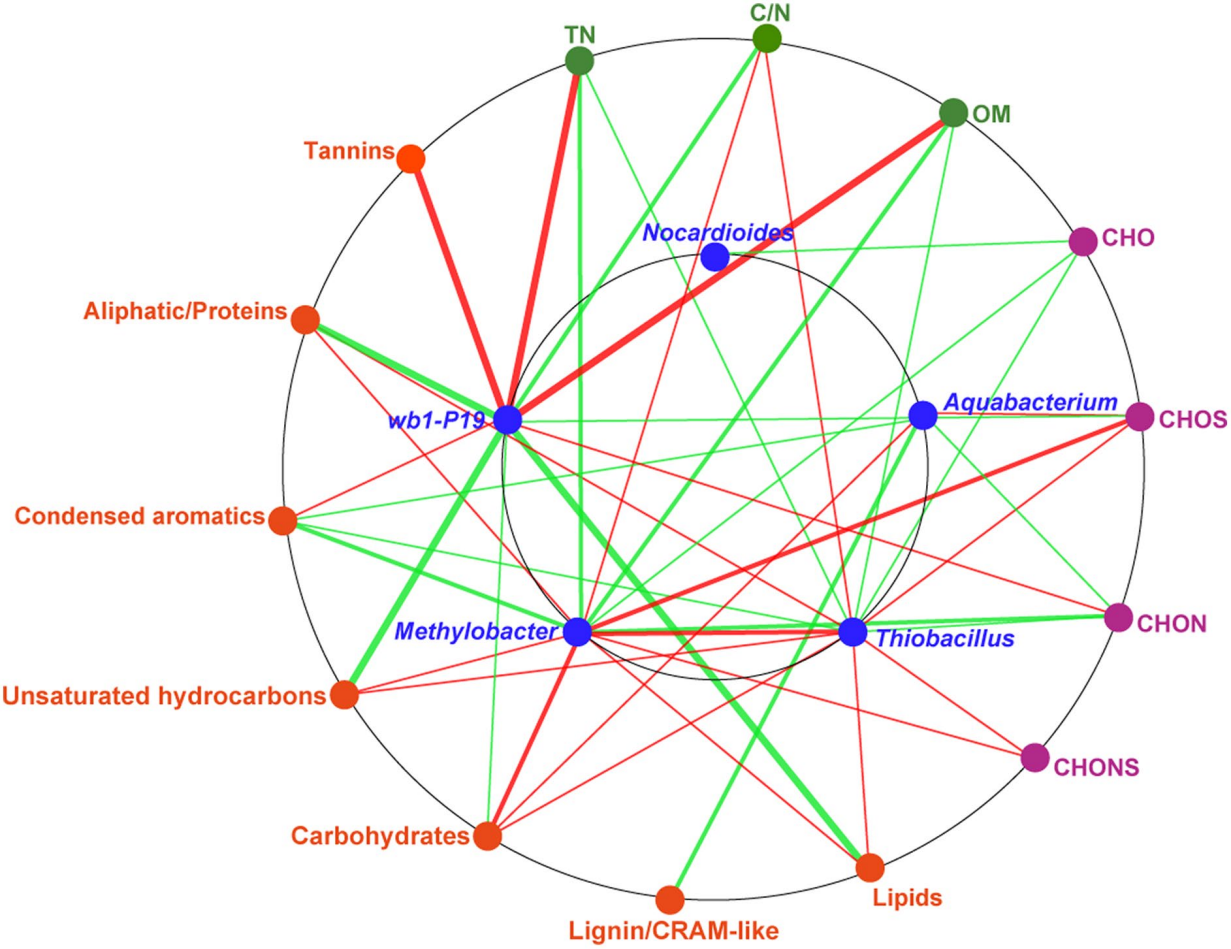
Network analysis based on the co-occurrence of the molecular composition of DOM, chemical properties, and key bacterial genera. The seven biochemical classes in the DOM are shown in orange ellipses; the four DOM subcategories are shown in purple ellipses; the chemical properties are shown in green ellipses; the major bacteria, including *Nocardioides*, *Aquabacterium*, *wb1-P19*, *Methylobacter*, and *Thiobacillus* are shown in blue ellipses. A connection represents a significant correlation (*p* < 0.05) according to Spearman’s analysis. The red lines denote positive correlations and the green lines denote negative correlations (the darker the color, the stronger the correlation).

To assess the soil environmental driving factors in bacterial genera, network analysis was conducted between DOM molecular composition, chemical properties, and bacterial genera (Figure 6). The chemical properties, including TN, OM, and C/N, were found to be significantly correlated with *wb1-P19*, *Methylobacter*, and *Thiobacillus*. TN and OM were strongly positively correlated with the genus *wb1-P19*. TN and OM in the soil were identified as nutrients and source of electron donors for microorganisms (Attard et al., 2010; Dong et al., 2020). C/N was positively related to *Methylobacter* and *Thiobacillus*. These results indicate that the CC soil C/N ratio was sufficiently beneficial for those two bacterial genera. At the same time, molecules (CHOS and CHONS) and compounds (unsaturated hydrocarbons, carbohydrates, lipids, and aliphatic/proteins) that contained most newly produced compounds in CC7Y were all positively related to *Methylobacter* and *Thiobacillus* (Figures 4C,D, 6 and Supplementary Figure S3). Additionally, a positive correlation was found between *Methylobacter* and *Thiobacillus*. Previous research showed that the positive correlations between DOM and microorganisms are indicative of biodegradation (Zhou et al., 2019; Li et al., 2021); here, the positive correlations may be interpreted as evidence that *Methylobacter* and *Thiobacillus* have a strong ability to degrade unsaturated hydrocarbons, carbohydrates, lipids, and aliphatic/proteins in soils after 7 years of CC.

## 4 Conclusion

This study has revealed the changes in soil chemical properties, DOM chemical diversity, microbial-community structure and their interactions in continuous cropping soil of tobacco. In CC soil, most of the soil chemical properties and the plant-derived DOM compounds (such as tannins) decreased significantly, the microbially derived DOM molecules aliphatic/proteins increased with increasing duration of CC in tobacco. Nevertheless, the DOM was high in compounds with H/C ratios >1.5, and low in compounds with O/C ratios <0.1, were produced preferentially in soil after 7 years of CC. The changes in microbial community structure reflect strong interaction with DOM over the process of CC. Key bacterial genera, such as *Methylobacter* and *Thiobacillus*, significantly influenced the DOM composition according to the duration of CC. These results have important guiding significance for the relationship between DOM and soil microbial communities during the CC of tobacco. In the future, the micro-biological degradation and transformation of DOM plays an important role in CC obstacles and should not be ignored.

## Data availability statement

The datasets presented in this study can be found in online repositories. The data presented in the study are deposited in the National Center for Biotechnology Information (NCBI) repository, accession number PRJNA1056661.

## Author contributions

PC: Data curation, Formal analysis, Funding acquisition, Writing – original draft. LW: Formal analysis, Investigation, Writing – original draft. WL: Data curation, Writing – original draft. XZ: Investigation, Writing – original draft. HG: Investigation, Writing – original draft. XZ: Funding acquisition, Investigation, Writing – original draft. QZ: Project administration, Resources, Writing – original draft. JL: Software, Writing – original draft. XL: Funding acquisition, Writing – original draft. AZ: Funding acquisition, Writing – review & editing.

## Footnotes

^1^www.majorbio.com
