# Supplementary figures and images for: Micro-biological degradation and transformation of dissolved organic matter following continuous cropping of tobacco
Source: Front Microbiol. 2024 Jan 26;15:1319895. doi: 10.3389/fmicb.2024.1319895 (PMC10853430; doi:10.3389/fmicb.2024.1319895)

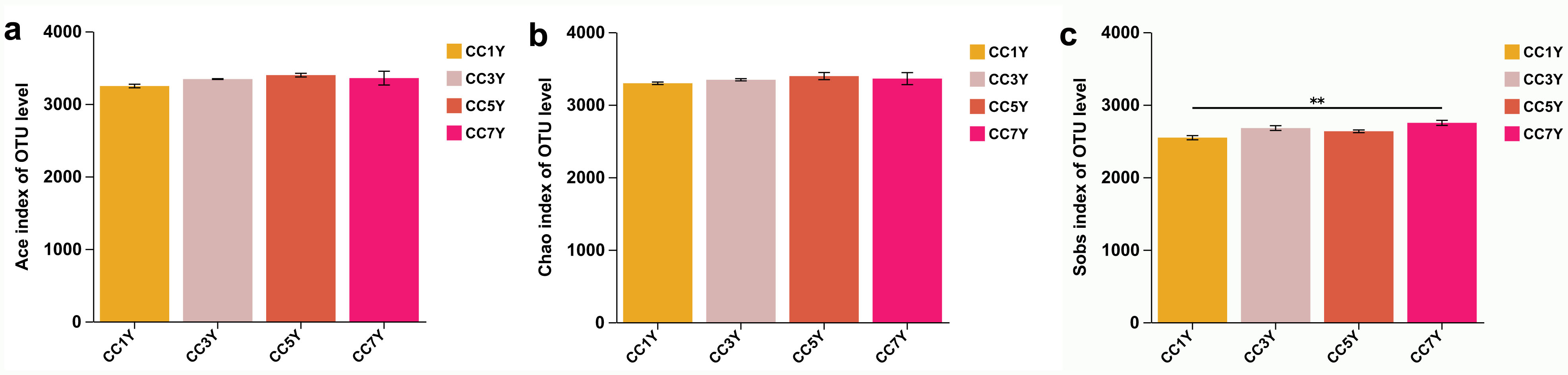

Supplement: Supplementary file 1 [file Image_1.JPEG]

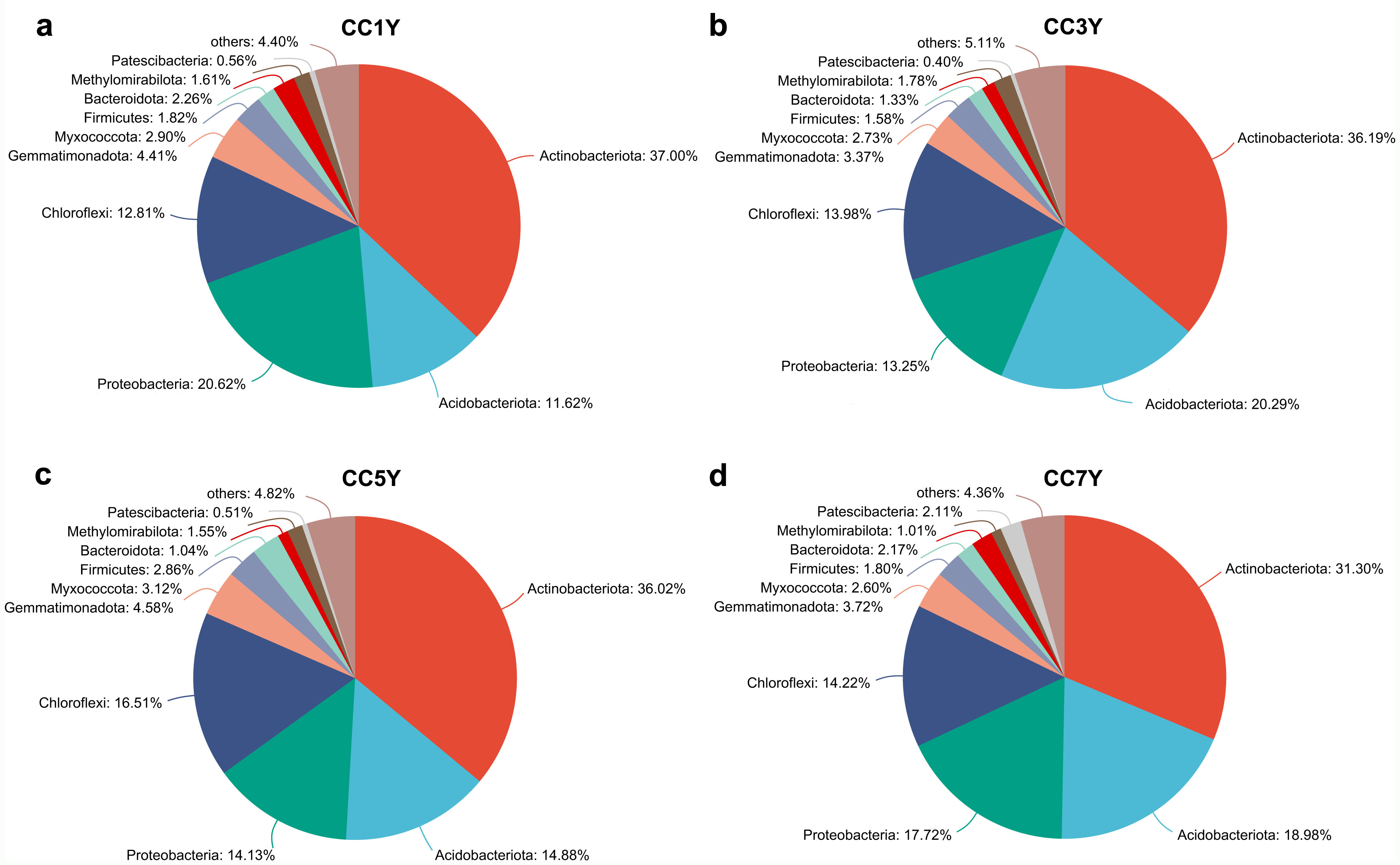

Supplement: Supplementary file 2 [file Image_2.JPEG]

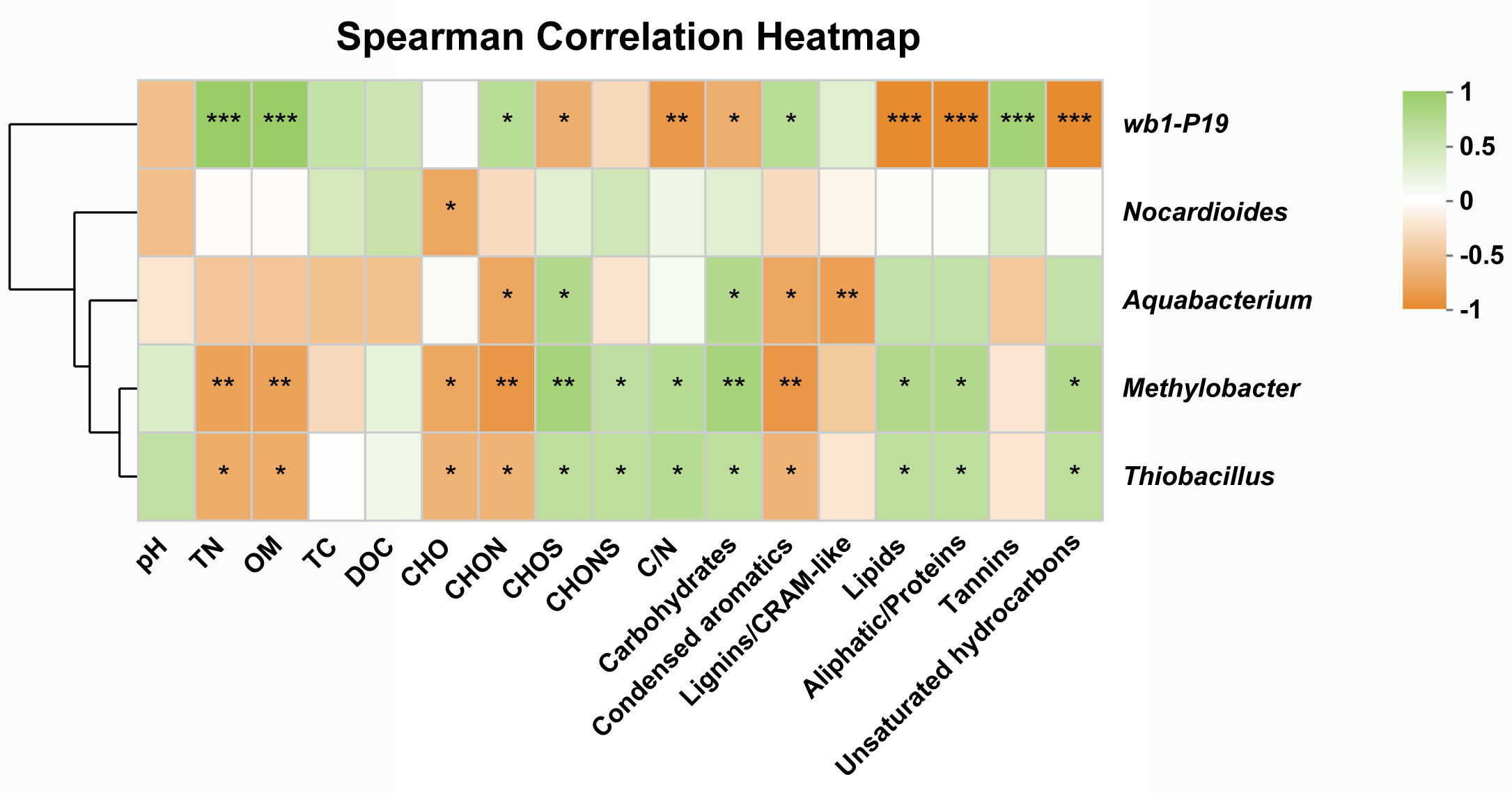

Supplement: Supplementary file 3 [file Image_3.JPEG]
